# Lynch syndrome or hereditary non polyposis colorectal cancer (HNPCC) in a moroccan family: Case report

**DOI:** 10.1016/j.amsu.2021.01.017

**Published:** 2021-01-17

**Authors:** F.Z. Outtaleb, A. Alami, N. Serbati, N. Benchakroun, Z. Bouchbika, H. Jouhadi, N. Tawfiq, S. Sahraoui, A. Benider, H. Dehbi

**Affiliations:** a Laboratory of Medical Genetics, Ibn Rochd University Hospital of Casablanca, Morocco; b Mohamed VI Oncology Center, Ibn Rochd University Hospital of Casablanca, Morocco; c Cellular and Molecular Pathology Laboratory, Casablanca Faculty of Medicine and Pharmacy, Hassan II University, Morocco

**Keywords:** Lynch syndrome, Oncogenetic consultation, MisMatch repair genes

## Abstract

**Introduction and importance:**

Colorectal cancer is a major global health problem. In 5% of cases, a genetic predisposition to cancer's syndrome is the etiology, such as Lynch syndrome. The population prevalence of Lynch syndrome has been estimated at 1/440. The objectives of this study are to show the interest of the oncogenetic consultation in the management of patients with suspicion of Lynch syndrome.

**Case presentation:**

It is a 70-year-old patient with a family history of different neoplasms. The patient has also been followed for an adenocarcinoma of the colon. An oncogenetic consultation was indicated, which led to the diagnosis of Lynch syndrome, according to the Amsterdam II criteria. A study of the MisMatch Repair genes was requested, to allow a pre-symptomatic diagnosis of apparented subjects at risk, and thus to also allow monitoring and early diagnosis of neoplasms or prophylactic measures.

**Discussion:**

Lynch syndrome is one of the most common cancer susceptibility syndromes. A constitutional deleterious mutation in one of the DNA MisMatch Repair genes, is responsible for nearly 70% of cases of this syndrome. The oncogenetic consultation and the identification of the genetics cause, makes it possible to set up specific monitoring and to offer a pre-symptomatic test to all major relatives of the index case.

**Conclusion:**

This medical observation shows the benefit of the oncogenetic consultation, if a genetic predisposition to cancer's syndrome is suspected. The diagnostic of this predisposition and monitoring of the propositus and his exposed, like in Lynch syndrome will help in the early management of cancers, specially colorectal cancer and endometrial adenocarcinoma.

## Highlights for review

• Lynch syndrome in a moroccan family: A case report:
• It shows the interest of a oncogenetic consultation if we suspect a hereditary predisposition to cancers.
• And the necessity of the surveillance of the propositus and his exposed relatives, to make possible an early management of cancers.

## 1 Introduction

Colorectal cancer (CRC) is a major global health problem, it is the third cancer in the world after lung cancer and breast cancer [1]. In Morocco, CRC is also the third most frequent tumor, the most common cancers in men being successively lung, prostate and colorectal cancer. While in women, the most frequent cancers are successively breast cancer, followed by cervical cancer and colorectal cancer, with a mortality of 12% in men and 7.1% in women [3], cancer being the second cause of death in our country after cardiovascular disease [2].

In 5% of cases of CRC, a genetic predisposition to cancer's syndrome is responsible of the disease, such as Lynch syndrome, the most common hereditary CRC, responsible of 3% of cases of CRC, and other tumors, specially endometrial cancer [4]. The population prevalence of Lynch syndrome has been estimated at 1/440 [5]. Lynch syndrome results from an inherited germline mutation responsible of an accelerated process of carcinogenesis, due to mismatch repair gene mutations [6].

The objectives of this case report are to show the Interest of the oncogenetic consultation and the benefit of a family investigation, in the management of patients with suspicion of hereditary form of CRC.

## 2 Patient and methods

This is a case report about a patient followed at the Ibn Rochd university hospital in Casablanca, for a colonic adenocarcinoma, suggesting Lynch syndrome, based on the Amsterdam II criteria (Table 1) [7], and tumoral spectre of Lynch syndrome (Table 2) [8]. This work has been reported in line with the SCARE 2020 criteria [9].

**Table 1:** Amsterdam II criteria [7].

Amsterdam II criteria [7]
- Colorectal or narrow spectrum cancers of HNPCC syndrome (endometrium, urothelium, small intestine) diagnosed in at least 3 relatives, - An affected individual related in the first degree to the other 2, - Affection of at least 2 successive generations, - Diagnosis at an age of less than 50 years in at least one of the affected subjects, - Exclusion of the diagnosis of familial adenomatous polyposis (FAP)

**Table 2:** Tumoral spectrum of Lynch syndrome |8].

Types of Lynch Syndrome Cancers:	
Narrow spectrum - Colorectal adenocarcinoma - Endometrial adenocarcinoma - Adenocarcinoma of the small intestine - Carcinoma of the upper urinary tract	
Narrow spectrum - Adenocarcinoma of the stomach - Ovarian adenocarcinoma - Bile duct adenocarcinoma - Glioblastoma - Sebaceous carcinoma	

## 3 Case presentation

**Fig. 1:**
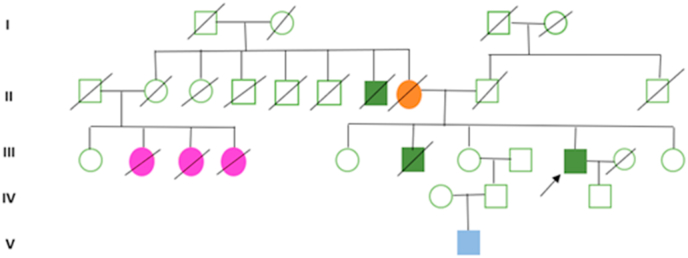
Genealogical tree of the family showing cases of colorectal cancers (in green), narrow spectrum cancers of Lynch syndrome: 3 cases of endometrium cancers (in pink), a case of large spectrum cancers: a cerebral tumor (in blue) in a 8 years-old child, and a case died at the age of 55 years-old of peritoneal carcinoma of undetermined origin (in orange). The genealogical tree shows also the affection of 2 successive generations.

It is a 70-year-old patient with a personal history of diabetes mellitus, dyslipidemia, ischemic heart disease and cholecystectomy. He has also a family history of different neoplasms (Fig. 1), as a brother died at the age of 50 from colorectal cancer (CRC) diagnosed at the age 45, 3 maternal cousins, first degree, died between the age of 50 and 60 years of gynecological cancer, and a maternal uncle, who died from a CRC.

The patient has also been followed since the age of 65 for a differentiated adenocarcinoma of the left colon, revealed by an occlusive syndrome, and treated by left colectomy and curage ganglionnaire and chemotherapy, and complicated one year ago by pulmonary metastasis, treated by stereotaxic radiotherapy. The colonoscopy surveillance has revealed two non-cancerous polyps of the colon.

In view of the suspicion of an inherited form of colorectal cancer, an oncogenetic consultation was indicated, which led to the diagnosis of Lynch syndrome, according to the Amsterdam II criteria. Subsequently, a study of the MMR genes was requested, in order to identify the germinal mutation responsible of the syndrome, and allow a pre-symptomatic diagnosis of apparented subjects at risk by detecting this mutation, and thus to allow monitoring and early diagnosis of neoplasms of Lynch spectrum, specially CRC and endometrial carcinoma.

## 4 Discussion

Lynch syndrome is one of the most common genetic predisposition to cancer's syndrome, Individuals with Lynch syndrome have 50%–70% lifetime risk of colorectal cancer, 40%–60% risk of endometrial cancer, and increased risk of several other malignancies. This syndrome is characterized by an autosomal.

### 4.1 Genetics of Lynch syndrome and the MisMatch repair system

Lynch syndrome is one of the most common genetic predisposition to cancer's syndrome, Individuals with Lynch syndrome have 50%–70% lifetime risk of colorectal cancer, 40%–60% risk of endometrial cancer, and increased risk of several other malignancies. This syndrome is characterized by an autosomal dominant inheritance, with high penetrance (about 85%) [6].

In Lynch syndrome, a constitutional deleterious mutation in one of the DNA MisMatch Repair genes (MMR), is responsible for nearly 70% of cases of this syndrome. The causes of the other cases are not known. The genes of the MMR system (MSH2, MLH1, MSH6 and PMS2) code for proteins that are involved in repairing DNA mismatches. Those proteins act in the form of heterodimers [10].

About 90% of alterations in the MMR system are constitutional mutations of the MSH2 (40%) or MLH1 (50%) genes. Germline mutations in MSH6 and in PMS2 are also described in about 10% of cases [11].

Two subgroups of Lynch syndrome have recently been identified, linked to constitutional biallelic mutations in an MMR gene [12]:

➢ ***The CMMRD syndrome (constitutional mismatch repair deficiency):***

This entity was recently described. It is caused by biallelic mutations in one of the MMR genes (MLH1, MSH2, MSH6 or PMS2).

The tumors are mainly located in the brain (high grade gliomas) and colorectal. An aspect of type 1 neurofibromatosis is also present with cutaneous manifestations, malignant hemopathies (acute leukemia, lymphomas) and rhabdomyosarcomas [13].

➢ MSH3 gene:

It is caused by homozygous constitutional mutations of the MSH3 gene. Studies on larger series of patients are currently underway to understand this entity [14].

## 4.2 Clinical presentation of Lynch syndrome and Amsterdam Criteria II

Clinically, Lynch syndrome is defined by the Amsterdam Criteria II (Table 1) [7]. Using these revised criteria, the sensitivity increases to 80%, but the specificity becomes less than 50% [15].

Thus some patients can validate these clinical criteria, without microsatellite instability or an abnormality of expression of MMR proteins. And without a mutation of an MMR genes has been demonstrated. “Syndrome X predisposition to colorectal cancer” [15] is the termz used to describ this constatio.

## 4.3 Bethesda criteria and orientation tests in tumor biopsy (somatic analysis)

There are criteria, called Bethesda criteria (Table 3) [7], which are used to determine whether tumors should be analyzed by orientation tests [17,18]. Those pre-screening or orientation tests are performed only on tumoral biopsys from subjects with cancer of the tumor spectrum.

**Table 3:** Bethesda criteria revised in 2004 [7], used to determine whether tumors should be analyzed by pre-screening techniques.

Bethesda criteria revised in 2004 [7]:
- Colorectal cancer diagnosed at an age of less than 50 years - Colorectal cancer diagnosed in an individual with a personal history of colorectal cancer or HNPCC spectrum, synchronous or metachronous, regardless of age at diagnosis - Colorectal cancer with suggestive pathological features (low degree of differentiation, “medullary” type architecture, dense lymphocytic infiltration of the tumor stroma) diagnosed at an age of less than 60 years - Colorectal cancer diagnosed in an individual with at least one first-degree relative with HNPCC spectrum cancer diagnosed at an age of less than 50 years - Colorectal cancer diagnosed in an individual with at least 2 first or second degree relatives with HNPCC spectrum cancer regardless of age at diagnosis.

Two principale techniques are used

➢ ***The immunohistochemistry***

This technique is used to study the tissue expression of proteins of the MMR system in tumor biopsy. The principle of this test is to look for a loss of expression of one or more of the proteins of the MMR system, within tumor cells compared to an internal control, usually normal colonic mucosa [19].

➢ ***The RER or MSI phenotype by PCR:***

The MSI (Micro Satellite Instability) phenotype, formerly called the RER (Positive Replication ERror) phenotype, corresponds to instability of microsatellites. These microsatellites are DNA sequences made up of patterns of one to five nucleotides repeated 10 to 20 times on average. Replication errors result in a change in the length of the microsatellites, and successive errors usually result in a shortening of the size of the microsatellites [19].

About 15% of all CRC cases have MSI status without constitutional alteration of the MMR system. In this case, it is a somatic alteration. The mechanism involved in the development of these cancers is methylation of the promoter of the MLH1 gene linked to senescence [20]. Those sporadic cancers are charterized using two tests:

***-The study of methylation of the MLH1 gene promoter:*** by a mechanism of senescence (aging) of the colonic mucosa in sporadic cancers.

***-The study of BRAF gene mutation:*** which is absent in CRC of the MSI phenotype of Lynch syndrome [19,21].

The search for tumor instability (MSI test) and the immunohistochemistry of MMR proteins are two orientation tests, making it possible to provide arguments for or against a continuation of the analysis of the genetic heritage:

*-* ***Immunohistochemistry and MSI test negative:*** the probability of Lynch syndrome is less than 5%;

***- Immunohistochemistry and/or positive MSI test***: the probability of Lynch syndrome is considerably increased. In this context, an anomaly is identified in almost 30% of cases if the family does not meet the Amsterdam criteria, and up to 90% if these criteria are met [16].

When the pre-screening tests are positive, the constitutional analysis of the MMR genes, ordered during an oncogenetic consultation is indicated.

### 4.4 Oncogenetics consultation and constitutional genetics analysis

The oncogenetic consultation is selected from the outset for all patients meeting the Amsterdam II criteria, as well as subjects with cancer of the Lynch spectrum, for whom the pre-screening tests are positive. If loss of expression of the MLH1 protein is demonstrated, the patient should be less than 60 years old.

Currently, it is recommended that the MSH2 and MLH1 genes be analyzed first for point mutations. If no mutation is identified, the analysis continues with the study of the MSH6 gene, since it represents the third MMR gene involved in this syndrome.

In the absence of identified mutations, the search for a complex anomaly (large deletion or insertion) of the MMR genes is undertaken. A large deletion is understood to mean the loss of one or more exons or even an entire gene. Large deletions are not detectable during sequencing. Indeed, the wild allele being present, all the exons are amplified by Polymerase Chain Reaction and sequenced, which masks the anomaly of the other allele.

Two outcomes are possible after the genetics analysis:

- No clearly deleterious mutation has been identified:

A genetics predisposition cannot be certainly eliminated, due to the technological limits, and the possible involvement of other genes unknown to date. Surveillance is then offered to all close relatives without knowing which subjects are really at risk. This monitoring is adapted according to the history of each family.

- A clearly deleterious mutation is identified:

This makes it possible to set up specific monitoring and to offer a pre-symptomatic test to all major relatives of the index case [16]. If the pre-symptomatic test detect the mutation responsible of Lynch syndrome in one of the relatives, regular colonoscopic surveillance and preventive hysterctomy after realisation of the sibling project are proposed.

In the case reported in this article, the constitutional analysis of the MMR genes has not been performed, due to the difficulty to access to the MMR gene analysis. The diagnosis of Lynch syndrome was based on the Amsterdam II criteria. In this case, the surveillance is offered to the propositus and all his relatives, specially to detect early CRC and endometrial cancers, by regular colonoscopic surveillance and gynecological surveillance, with a colonoscopy every 1–2 years for CRC screening and endometrial swab with endovaginal ultrasound every 1–2 years for endometrial cancer screening.

## 5 Conclusion

In Morocco, cancer is a major public health problem. The management of this pathology is generally done late, because the diagnosis is still frequently made at an advanced stage is often made at an advanced stage. Therfore, the diagnostic of genetic predisposition to cancer's syndrome, and monitoring of the propositus and his exposed relatives, like in Lynch syndrome will help in the early management of cancers, specially CRC and endometrial adenocarcinoma.

## Ethical approval

None.

## Funding

There is no sources of funding.

## Author contribution

Fatima Zahra Outtaleb: Corresponding author, writing the paper.

The guarantor is that individual who accepts full responsibility for the work and/or the conduct of the study, had access to the data, and controlled the decision to publish.

A. Alami: writing the paper.

N. Serbati: correction of the paper.

N. Benchakroun: correction of the paper.

Z. Bouchbika: correction of the paper.

H. Jouhadi: correction of the paper.

N. Tawfiq: correction of the paper.

S. Sahraoui: correction of the paper.

A. Benider: correction of the paper.

H. Dehbi: correction of the paper.

## Consent

Written informed consent was obtained from the patient for the publication for the publication this case report. A copy of the written consent is available for review by the Editor-in-Chief of this journal on request.

## Registration of Research Studies

Name of the registry: http://www.researchregistry.com

Unique Identifying number or registration ID: researchregistry6394

Hyperlink to your specific registration (must be publicly accessible and will be checked): https://www.researchregistry.com/browse-the-registry#home/

## Guarantor

The Guarantor is the one or more people who accept full responsibility for the work and/or the conduct of the study, had access to the data, and controlled the decision to publish.

Fatima Zahra Outtaleb

## Patient consent

Written informed consent was obtained from the patient for the publication for the publication this case report. A copy of the written consent is available for review by the Editor-in-Chief of this journal on request.

## Provenance and peer review

Not commissioned, externally peer-reviewed.

## Declaration of competing interest

The authors declare having no conflicts of interest.
